# Gesundheitskompetenz und Notfallverhalten

**DOI:** 10.1007/s10049-021-00859-z

**Published:** 2021-03-25

**Authors:** Markus Wehler, Anja Kalch, Helena Bilandzic, Thomas Händl

**Affiliations:** 1 Zentrale Notaufnahme und IV. Medizinische Klinik, Universitätsklinikum Augsburg, Stenglinstraße 2, 86156 Augsburg, Deutschland; 2 Institut für Medien, Wissen und Kommunikation, Universität Augsburg, Augsburg, Deutschland; 3 Zentrale Notaufnahme, Klinikum Garmisch-Partenkirchen, Garmisch-Partenkirchen, Deutschland

**Keywords:** Ressourcenverbrauch, Notaufnahme, Patienten mit nichtdringlichem Behandlungsbedarf, Querschnittsbefragung, Motive, Ressource utilisation, Emergency department, Patients with nonurgent conditions, Cross-sectional survey, Motivations

## Abstract

**Hintergrund:**

Die Anzahl an Patienten in Notaufnahmen steigt seit Jahren an. Im Besonderen wächst der Anteil an Patienten mit einem nichtdringlichen Behandlungsbedarf. Bislang ist unklar, ob dies in Deutschland auch auf eine eingeschränkte Gesundheitskompetenz zurückgeführt werden kann.

**Ziel der Arbeit:**

Ziel der Studie war es, die Gesundheitskompetenz (GK) von Notfallpatienten mit nichtdringlichem Behandlungsbedarf zu erfassen, mit allgemeinen Bevölkerungsdaten zur GK zu vergleichen und Zusammenhänge mit der subjektiv empfundenen notfallmedizinischen Versorgung zu betrachten.

**Material und Methode:**

Es wurde eine Querschnittsbefragung in der zentralen Notaufnahme und der angeschlossenen kassenärztlichen Bereitschaftspraxis (KVB-Praxis) des Universitätsklinikums Augsburg durchgeführt. Insgesamt wurden 448 Patienten mit nichtdringlichem Versorgungsbedarf befragt.

**Ergebnisse:**

Es zeigt sich, dass die GK der Notfallpatienten schlechter ausfällt als die der deutschen Gesamtpopulation. Patienten mit niedriger GK nehmen eine höhere subjektive Behandlungsdringlichkeit wahr, kennen signifikant seltener alternative Anlaufstellen zur Behandlung und möchten nach hausärztlicher Versorgung häufiger in der Notaufnahme oder der KVB-Praxis eine zweite Meinung einholen.

**Diskussion:**

Die Ergebnisse zeigen einen Handlungsbedarf für eine Verbesserung der Gesundheitskompetenz auf. Dabei sind insbesondere die aktuellen strukturellen Veränderungen der Notfallversorgung in Deutschland zu berücksichtigen.

## Abstract (en)

**Background:**

The number of patients with nonurgent health conditions using emergency departments (ED) is growing steadily. It is unclear however whether this is associated with limited health literacy of patients.

**Objectives:**

This study aims to explore the health literacy of patients with nonurgent conditions in an ED, compare it with the health literacy of the general population and identify relationships between health literacy and patients’ views on emergency care.

**Materials and methods:**

A total of 448 patients with nonurgent conditions participated in a cross-sectional survey in the ED of a German university hospital.

**Results:**

The study shows that health literacy of nonurgent emergency patients is significantly lower than the health literacy of the German population. Patients with lower levels of health literacy perceive higher treatment urgency, are significantly less informed about treatment options other than the ED and are more likely to visit the ED or the outpatient walk-in practice for a second opinion after having seen their general practitioner.

**Conclusion:**

The results indicate a need for action to improve health literacy in Germany. In particular, current structural changes in emergency care in Germany have to be considered.

Patienten mit nichtdringlichem Behandlungsbedarf stellen eine Herausforderung für Notaufnahmen in Deutschland dar. Bislang sind zwar allgemeine Motive für das Aufsuchen der Notaufnahmen bekannt, die zugrunde liegenden Ursachen jedoch nicht. Der vorliegende Beitrag analysiert die Gesundheitskompetenz (GK) als relevante Einflussgröße für das Notfallverhalten nichtdringlicher Patienten. Ein genaueres Verständnis des Verhaltens dieser Patienten ist essenziell für eine Entlastung der Notaufnahmen.

Die Notaufnahmen in Deutschland waren jahrzehntelang mit einem stetig wachsenden Patientenaufkommen konfrontiert, im Besonderen von Patienten, die im medizinischen Sinne nicht als akuter Notfall gelten [18, 20, 21]. Vorliegende Studien aus Patientenperspektive zeigen bereits, dass neben Terminaspekten bei Haus- oder Fachärzten und zeitlicher Flexibilität vor allem die in der Notaufnahme erwartete qualitativ höherwertige und multidisziplinäre Versorgung als Motiv genannt wird, ebenso wie Unsicherheit und Angst über den eigenen Gesundheitszustand [18, 20, 21, 23]. Die Coronapandemie hat diesen Trend im Bereich der Selbsteinweisungen durch Patienten mit niedriger Behandlungsdringlichkeit umgekehrt [2, 10, 14, 24, 27]. Viele Patienten verzichten derzeit eher auf eine Selbsteinweisung in der Notaufnahme aus Angst vor einer Ansteckung mit dem Coronavirus oder möglichen Versorgungsengpässen [14].

Ängste und Unsicherheiten über die eigene Gesundheit scheinen damit in beiden Entwicklungen eine relevante Rolle für das Notfallverhalten bei Patienten mit nichtdringlichem Versorgungsbedarf einzunehmen. Da wenig Zweifel daran besteht, dass nach dem Ende der Coronapandemie wieder mit einem Zuwachs nichtdringlicher Patienten zu rechnen ist [10, 13], erscheint eine genauere Untersuchung, die über die Nennung von Motiven hinausgeht, dringend erforderlich. Dies gilt umso mehr, da die geplante Strukturreform durch die Coronapandemie vorerst gebremst ist [3].

Die vorliegende Studie analysiert dazu die GK von Patienten mit nichtdringlichem Behandlungsbedarf. Die GK steht generell im Zusammenhang mit einer Vielzahl gesundheitlicher Parameter [12]. Eine Analyse der GK von Notaufnahmepatienten existiert bisher jedoch nur für den angloamerikanischen Raum. Dort zeigt sich eine häufigere Nutzung von Notaufnahmen durch Patienten mit niedriger GK und einem gleichzeitig geringeren Zugang zu Hausärzten [1, 5]. Allerdings kann diese Evidenz aufgrund der strukturellen Unterschiede des Gesundheitswesens nicht direkt auf Deutschland übertragen werden. Ziel der vorliegenden Studie ist deshalb die Erfassung der GK von Notaufnahmepatienten in Deutschland, ein Vergleich zur Verteilung der GK in der allgemeinen Bevölkerung [9, 16] sowie die Analyse von empirischen Zusammenhängen zwischen GK, soziodemografischen und gesundheitsbezogenen Parametern.

## Methode

### Datenerhebung

Die Studie wurde im vierten Quartal 2017 und ersten Quartal 2018 über einen Zeitraum von 10 Wochen als Querschnittsbefragung der Patienten der zentralen Notaufnahme des Universitätsklinikums Augsburg (ZNA) und im Wartebereich der angeschlossenen kassenärztlichen Bereitschaftspraxis (KVB-Praxis) durchgeführt. Die Ersteinschätzung erfolgte dabei für alle Studienpatienten in der ZNA. Die Befragung wurde an Werktagen zwischen 10 und 14 Uhr sowie zwischen 16 und 20 Uhr durchgeführt. Nachtzeiten sowie Wochenenden und die Weihnachtsferien wurden ausgespart, da Patienten zu diesen Zeiten über wenig Alternativen bei der Versorgung verfügen. Alle Befragten füllten nach einer Aufklärung und schriftlichen Einwilligung den Fragebogen in einer von drei verfügbaren Sprachen (Deutsch, Russisch, Türkisch) selbstständig auf Tablets aus.^1^ Die Studie wurde von der Ethikkommission des Universitätsklinikums positiv beurteilt (Bearb.-Nr. 201731).

### Stichprobe

Zur Zielpopulation der Studie zählten alle Patienten über 18 Jahre, die gemäß den Emergency-Severity-Index-Kategorien (ESI) 4 und 5 [6] einen nichtdringlichen Behandlungsbedarf aufwiesen – unabhängig von der Art der Einweisung. Ausgeschlossen wurden Patienten, die keine Einwilligung zur Studienteilnahme abgaben, den Fragebogen nicht selbstständig ausfüllen konnten, wegen Kommunikationsproblemen nicht an der Befragung teilnehmen konnten oder die ohne Wartezeit direkt behandelt bzw. weiterverwiesen wurden. Insgesamt nahmen 475 Patienten an der Befragung teil. 27 Personen wurden vor der Datenanalyse ausgeschlossen, da sie weniger als 60 % der Fragen beantwortet hatten. Gemessen an der Gesamtzahl aller Patienten mit ESI-Kategorie 4 und 5 während der Befragungszeit im Klinikum (*N* = 1579) entspricht dies einem Anteil von 30,08 %.

### Fragebogen

Die GK wurde mit der deutschen Version der HLS-EU-Q16-Kurzskala [15] erfasst, die in dieser Form auch für die deutsche Bevölkerungsstudie [9] verwendet wurde. Die 16 Items erfassten die subjektive Schwierigkeit, mit gesundheitsspezifischen Anforderungen umzugehen (4-stufige Skala von „sehr schwierig“ bis „sehr einfach“). Die Einteilung der GK-Stufen erfolgte nach dem in der Literatur empfohlenen Verfahren [15]: Die Items wurden dichotomisiert („sehr einfach“ und „ziemlich einfach“ = 1, „ziemlich schwierig“ und „sehr schwierig“ = 0) und anschließend in einem Summenscore zusammengefasst. Ein Score zwischen 13 und 16 wurde als ausreichende GK kategorisiert, von 9 bis 12 als problematische GK und von 1 bis 8 als inadäquate GK [9, 15].

Als soziodemografische Faktoren wurden Alter, Geschlecht, Bildung und Migrationshintergrund erfasst. Das Alter wurde in drei Altersgruppen zusammengefasst (junge Patienten: 18 bis 39 Jahre, Patienten mittleren Alters: 40 bis 59 Jahre, ältere Patienten: 60 Jahre und älter; [9]). Die Bildung wurde mit der *International-Standard-Classification-of-Education-1997*-Skala (ISCED-97) erhoben und kategorisiert [9, 22]. Ein Migrationshintergrund wurde erfasst, wenn der Patient selbst oder die Eltern des Patienten nicht in Deutschland geboren wurden [16].

Um die Einweisung der Patienten zu erfassen, wurde erfragt, wie die Patienten in die Klinik gekommen sind (1 = „Notarzt und Rettungswagen“, 2 = „formale Einweisung des eigenen Arztes in die Klinik, nach direkter vorhergehender Untersuchung“, 3 = „Selbsteinweisung nach telefonischem Kontakt mit Praxis“, 4 = „Selbsteinweisung ohne Arztkontakt“). Die Kategorien 3 und 4 wurden für die Auswertung zu „selbsteinweisende Patienten“ zusammengefasst.

Die subjektive Behandlungsdringlichkeit wurde mit der Frage „Wie dringend schätzen Sie ihren Behandlungsbedarf selbst ein?“ ermittelt (5-stufige Antwortskala: 0 = „nicht dringlich“, 1 = „weniger dringlich“, 2 = „dringlich“, 3 = „sehr dringlich“, 4 = „akut“).

Zur Erfassung der hausärztlichen Versorgung wurden die Patienten gebeten anzugeben, ob sie einen Hausarzt haben (1 = „ja“, 0 = „nein“) und ob sie kurz zuvor schon bei diesem (oder einem anderen niedergelassenen Arzt) in Behandlung waren (1 = „ja“, 0 = „nein“).

Die Variable Beschwerdedauer unterscheidet akute Beschwerden (1 = „bis zu 24 h“) von länger andauernden Beschwerden (0 = „länger als 24 h“). Zur Erfassung der Motive wurden die Patienten gebeten, die genauen Gründe anzugeben, „wieso Sie heute in die Notaufnahme bzw. Kassenärztliche Bereitschaftspraxis gekommen sind“. Den Befragten wurden eine Liste aus elf Motiven sowie eine offene Antwortkategorie für sonstige Motive vorgelegt (Tab. 1; [7]). Es waren Mehrfachantworten möglich.

**Table 1:** 

Motive	Keine Alternativen bekannt		Verschlechterung Symptome		Zweite Meinung		Ärztlicher Verweis^a^		Bessere Versorgung	
	% (*n*)	OR (95 %-KI)	% (*n*)	OR (95 %-KI)	% (*n*)	OR (95 %-KI)	% (*n*)	OR (95 %-KI)	% (*n*)	OR (95 %-KI)
*GK-Level*										
Ausreichend	3 (4)	1,00 (Ref.)	28,6 (38)	1,00 (Ref.)	5,3 (7)	1,00 (Ref.)	39,8 (37)	1,00 (Ref.)	8,3 (11)	1,00 (Ref.)
Problematisch	11,9 (22)	4,03 (1,33–12,20)*	17,3 (32)	0,47 (0,27–0,81)**	2,7 (5)	0,51 (0,16–1,65)	43,2 (80)	1,75 (1,03–2,99)*	12,4 (23)	1,67 (0,76–3,68)
Inadäquat	11,8 (11)	4,11 (1,24–13,60)*	18,3 (17)	0,50 (0,25–0,99)*	14 (13)	3,22 (1,17–8,87)*	34,6 (46)	2,04 (1,03–4,02)*	18,3 (17)	2,54 (1,07–6,02)*
Aufnahmebereich	–	1,75 (0,81–3,77)	–	1,70 (0,97–2,97)	–	0,20 (0,04–0,93)*	–	0,06 (0,03–0,15)***	–	0,82 (0,38–1,76)
Alter	–	0,97* (0,95–1,00)	–	0,99 (0,98–1,01)	–	0,98 (0,96–1,02)	–	1,04 (1,03–1,06)***	–	1,00 (0,98–1,02)
Geschlecht	–	0,71 (0,34–1,47)	–	1,03 (0,62–1,71)	–	0,79 (0,33–1,93)	–	0,94 (0,58–1,52)	–	0,68 (0,36–1,29)
Bildung	–	1,14 (0,52–2,50)	–	0,84 (0,49–1,43)	–	0,80 (0,30–2,11)	–	0,76 (0,46–1,26)	–	0,75 (0,38–1,49)
Migrationshintergrund	–	1,68 (0,68–4,12)	–	1,56 (0,82–3,01)	–	2,59 (0,96–6,94)	–	0,66 (0,34–1,27)	–	0,96 (0,40–2,29)

Codierung der Variablen: Geschlecht (0 = männlich, 1 = weiblich), der Bildungsstand (0 = niedrige und mittlere Bildung, 1 = hohe Bildung), Migrationshintergrund (0 = kein Migrationshintergrund, 1 = Migrationshintergrund), Aufnahmebereich (0 = Notaufnahme, 1 = KVB-Praxis)

Für die Motive „Empfehlung durch Hotline“, „Empfehlung durch Freunde/Familie“, „kein Termin in Hausarztpraxis“ und „Hauarztpraxis heute geschlossen“ liegen keine statistisch relevanten Zusammenhänge mit dem GK-Level vor. Für die Motive „grundsätzlicher Besuch der Notaufnahme“ (*n* = 3) und „kein Arzt bekannt“ (*n* = 18) konnte aufgrund der zu geringen Fallzahl kein statistischer Zusammenhang überprüft werden

*OR* Odds Ratio, *KI*  Konfidenzintervall, *Ref.* Referenzkategorie, *GK* Gesundheitskompetenz, *KVB*-Praxis kassenärztliche Bereitschaftspraxis

*p < 0,05, **p < 0,01, ***p < 0,001

^a^Das Motiv „Ärztlicher Verweis“ bezieht sich auf die Selbstauskunft der Befragten und umfasst sowohl Patienten, die angaben, in die Klinik nach einer Untersuchung eingewiesen worden zu sein, als auch Selbsteinweiser, die z. B. angaben, eine telefonische Empfehlung von einer Praxis erhalten zu haben

### Statistische Analyse

Für den Vergleich der GK-Level in der Gesamtpopulation und der Notfallpatienten wurde ein χ^2^-Unabhängigkeitstest auf Basis der publizierten Häufigkeiten der Kurzskala berechnet [9]. Zur Überprüfung eines Zusammenhangs zwischen dem GK-Level und soziodemografischen Merkmalen wurden Kreuztabellen sowie weitere χ^2^-Unabhängigkeitstests berechnet. Zur Analyse des Zusammenhangs von GK und subjektiver Behandlungsdringlichkeit diente eine ANCOVA. Für den Zusammenhang von GK mit dem Einbezug der hausärztlichen Versorgung, der Symptomdauer sowie den Motivationen, die ZNA aufzusuchen, wurden logistische Regressionen gerechnet.

## Ergebnisse

Von den 448 befragten Patienten haben 415 den deutschsprachigen Fragebogen ausgefüllt, 19 den russischsprachigen Fragebogen und 14 den türkischsprachigen Fragebogen. Es wurden 334 Patienten im Wartebereich der ZNA und 114 Patienten im Wartebereich der KVB-Praxis befragt. Die Mehrheit der Patienten ist selbstständig oder mit Angehörigen in die Klinik gekommen (*n* = 252, 56,3 %), 114 Patienten gaben an, über eine formale Einweisung des eigenen Arztes zu verfügen (*n* = 114, 25,4 %), 36 Personen (8 %) gaben einen telefonischen Verweis an das Klinikum an und nur 13 Befragte (2,9 %) gaben an, mit dem Rettungsdienst gebracht worden zu sein, 33 Personen haben dazu keine Angabe gemacht (7,4 %).

Das Durchschnittsalter der Befragten lag bei 37,15 Jahren (SD = 16,42). Zur Verteilung der Soziodemografie siehe Tab. 2.

**Table 2:** 

	GK-Level			
	Ausreichend % (*n*)	Problematisch % (*n*)	Inadäquat % (*n*)	*n* gesamt
*Alter*				*413*
18–39 Jahre	31,9 (82)	44 (113)	24,1 (62)	258
40–59 Jahre	38,8 (40)	44,7 (46)	16,5 (17)	104
60 plus Jahre	25,5 (13)	49 (25)	25,5 (13)	51
*Bildung*				*397*
Niedrig	37,5 (18)	31,3 (15)	31,3 (15)	49
Mittel	30,9 (60)	46,9 (91)	22,2 (43)	194
Hoch	34,4 (53)	48,1 (74)	17,5 (27)	154
*Geschlecht*				*447*
Frauen	36,9 (86)	42,9 (100)	20,2 (47)	234
Männer	30,0 (64)	46 (98)	23,9 (51)	213
*Migrationshintergrund*				*409*
Mit MH	30 (18)	46,7 (28)	23,3 (14)	81
Ohne MH	33,8 (111)	44,8 (147)	21,3 (70)	328

*GK* Gesundheitskompetenz

**Figure 1:**
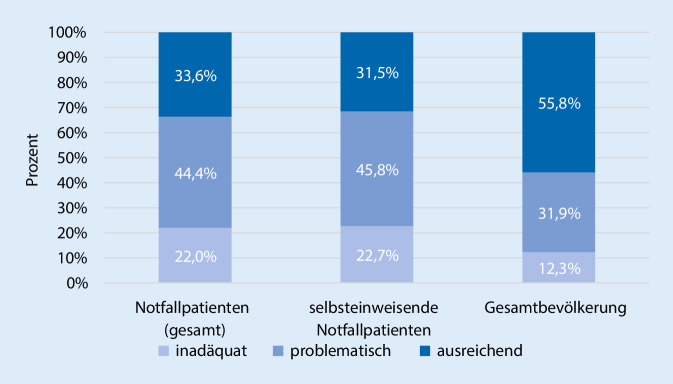

Der Vergleich der GK-Level zeigt, dass die GK der Notfallpatienten insgesamt schlechter ausgeprägt ist als in der Gesamtpopulation, *χ*^2^(2) = 85,70, *p* < 0,001, Cramers *V* = 0,13. Dies zeigt sich auch, wenn man nur die Gruppe der selbsteingewiesenen Patienten im Vergleich zur Gesamtpopulation betrachtet, *χ*^2^(2) = 68,14, *p* < 0,001, Cramers *V* = 0,11 (Abb. 1).

Es liegen keine signifikanten Unterschiede der GK-Levels in Abhängigkeit von Geschlecht, Alter, Migrationshintergrund oder der Bildung der Patienten vor (Tab. 2). Das GK-Level beeinflusst jedoch, welchen Versorgungsbereich ein Patient wahrnimmt. Patienten mit inadäquater GK gehen mit einer höheren Wahrscheinlichkeit in die ZNA statt in die KVB-Praxis im Vergleich zu Patienten mit ausreichender GK (Odds Ratio = 1,98, 95 %-Konfidenzintervall [1,05; 3,73], Wald (1) = 4,44, *p* = 0,35). Neben der klinischen Versorgung zeigt sich auch ein Zusammenhang der GK mit der Einbindung in die hausärztliche Versorgung: Patienten mit inadäquater und problematischer GK berichten häufiger von länger bestehenden (>24 h) Symptomen und haben ihren Arzt wegen dieser Symptome mit höherer Wahrscheinlichkeit bereits aufgesucht (Tab. 3). Es ist weniger wahrscheinlich, dass sie eine akute Verschlechterung der Symptome berichten im Vergleich zu Patienten mit ausreichender GK (Tab. 1). Dementsprechend geben diese beiden Patientengruppen auch mit höherer Wahrscheinlichkeit an, vom Arzt in die ZNA verwiesen worden zu sein (Tab. 1). Die Einbindung der hausärztlichen Versorgung ist jedoch bei Patienten mit inadäquater GK nicht optimal: Diese Patientengruppe gibt mit höher Wahrscheinlichkeit an, die ZNA für eine zweite Meinung aufgesucht zu haben und dort eine qualitativ bessere Versorgung als beim Hausarzt zu erwarten (Tab. 1). Ein Unterschied in der grundsätzlichen Verfügbarkeit eines Hausarztes liegt jedoch nicht vor (Tab. 3) – auch wenn Patienten mit problematischer und inadäquater GK eher angeben, keine Alternative in der Notfallsituation zu kennen (Tab. 1).

**Table 3:** 

	Hausarzt vorhanden (*n* = 383)		Hausarzt aufgesucht (*n* = 383)		Symptomdauer länger als 24 h (*n* = 383)	
	% (*n*)	OR (95 %-KI)	% (*n*)	OR (95 %-KI)	% (*n*)	OR (95 %-KI)
*GK-Level*						
Ausreichend	91,1 (123)	1,00 (Ref.)	43 (58)	1,00 (Ref.)	55,6 (75)	1,00 (Ref.)
Problematisch	93,1 (176)	1,15 (0,43–3,09)	55,6 (105)	1,78 (1,09–2,89)*	68,3 (129)	1,71 (1,05–2,77)*
Inadäquat	91,5 (86)	0,74 (0,25–2,23)	51,1 (48)	1,88 (1,03–3,42)*	72,3 (68)	1,92 (1,05–3,54)*
Aufnahmebereich	–	1,63 (0,57–4,68)	–	0,36 (0,21–0,61)***	–	1,36 (0,81–2,30)
Alter	–	1,05 (1,02–1,10)**	–	1,03 (1,02–1,05)***	–	1,01 (1,00–1,03)
Geschlecht	–	2,92 (1,16–7,32)*	–	0,89 (0,58–1,37)	–	0,63 (0,40–0,97)*
Bildung	–	0,59 (0,24–1,44)	–	0,91 (0,57–1,44)	–	0,57 (0,36–0,90)*
Migrationshintergrund	–	0,58 (0,21–1,61)	–	1,03 (0,57–1,86)	–	1,37 (0,73–2,56)

*Anmerkung.* Codierung der Variablen: Geschlecht (0 = männlich, 1 = weiblich), der Bildungsstand (0 = niedrige und mittlere Bildung, 1 = hohe Bildung), Migrationshintergrund (0 = kein Migrationshintergrund, 1 = Migrationshintergrund), Aufnahmebereich (0 = Notaufnahme, 1 = KVB-Praxis)

*OR* Odds Ratio, *KI* Konfidenzintervall, *Ref.* Referenzkategorie, *GK* Gesundheitskompetenz

*p < 0,05, **p < 0,01, ***p < 0,001

**Figure 2:**
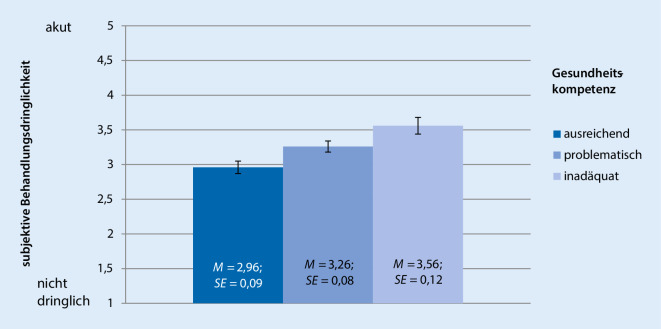

Im Einklang mit der stärkeren Nutzung des Notfallsystems nehmen Patienten mit inadäquater GK zudem eine höhere Behandlungsdringlichkeit wahr als Patienten mit problematischer GK (*p* = 0,034) oder ausreichender GK (*p* < 0,001; Abb. 2).

## Diskussion

Die vorliegende Analyse zeigt erstmals einen Zusammenhang zwischen der GK von Patienten, dem Aufsuchen einer Notfallversorgung sowie gesundheitsbezogenen Parametern in Deutschland. Vergleichbar mit den Ergebnissen aus US-amerikanischen Notaufnahmen [1, 5] haben auch die Patienten in der betrachteten deutschen ZNA sowie der benachbarten KVB-Praxis eine niedrigere GK als die Gesamtbevölkerung. Auch wenn die Zusammenhänge mit gesundheitsbezogenen Faktoren aufgrund der begrenzten Fallzahlen vorsichtig interpretiert werden müssen, weisen sie auf einen zweifachen Einfluss der GK hin: Einerseits zeigt sich, dass Patienten mit niedriger GK ihren eigenen Versorgungsbedarf dringlicher einschätzen; andererseits scheint es ihnen schwerer zu fallen, sich in dem komplexen deutschen Gesundheitssystem zu orientieren [11] und alternative Versorgungsmöglichkeiten zur Notfallversorgung zu finden.

Dies spricht grundsätzlich für die geplanten strukturellen Änderungen der Notfallversorgung. In Zukunft sollen integrierte Notfallzentren die Patienten in die adäquate Versorgungsebene lenken [11]. Dies entlastet nicht nur die Versorgungssysteme, sondern verringert auch die Entscheidungskomplexität der Patienten. Aus Sicht der Patienten stellt dies jedoch gleichzeitig auch eine Einschränkung der individuellen Entscheidungsfreiheit dar. Eine grundlegende Voraussetzung dafür, dass diese strukturelle Änderung von den Patienten, insbesondere Patienten mit niedriger GK, akzeptiert wird, ist die Bereitstellung umfassender und vor allem passgenauer Informationen zu den strukturellen Veränderungen und der Einbezug der Patienten in eine informierte Entscheidungsfindung. In einem zweiten Schritt ist eine entsprechende Förderung der GK anzustreben. Eine aktuelle Studie konnte zeigen, dass eine dreijährige webbasierte Informations- und Schulungsmaßnahme zur Verbesserung der Gesundheitskompetenz die Inanspruchnahme der Notaufnahme, die Hospitalisierungsrate wie auch die Gesundheitskosten pro Kopf relevant senken konnte [4].

### Limitationen

Die Stichprobe weist im Hinblick auf das Alter, den Bildungsstand und den Migrationsanteil leichte Abweichungen zur Gesamtbevölkerung in Deutschland auf. Vor allem der Anteil jüngerer Personen ist in der Stichprobe erhöht [25]. Das Alter steht jedoch weder hier noch in der Gesamtbevölkerung in Abhängigkeit zur GK [9]. Die Stichprobe weist zudem weniger niedrig gebildete Personen auf [26]. Diese spezifische Zusammensetzung der Stichprobe kann eine mögliche Ursache dafür sein, dass sich im Gegensatz zur Gesamtbevölkerung [9] kein Unterschied in Abhängigkeit vom Bildungstand zeigt. Allerdings finden auch andere Studien keinen [16] oder nur sehr schwache Zusammenhänge zwischen GK und Bildung [8]. Sowohl beim Alter als auch bei der Bildung ist zu berücksichtigen, dass die Tablet-gestützte Durchführung und die Befragungssituation in der ZNA möglicherweise ältere oder auch niedrig gebildete Patienten von der Teilnahme abgeschreckt haben.

Die Vergleichbarkeit mit den Daten der Gesamtbevölkerung wird zudem dadurch beschränkt, dass sich die Befragten hier in einer Notfallsituation befanden, wodurch eine andere Interpretation der GK-Items möglich ist.

### Ausblick

Politische Maßnahmen als Reaktion auf die steigenden Patientenzahlen in deutschen Notaufnahmen fokussieren strukturelle Veränderungen primär aus klinisch-ökonomischer Perspektive. Eine patientenorientierte Sicht wird dabei kaum eingenommen. Dabei erscheint es vor allem notwendig, die GK als relevanten Faktor für das Notfallverhalten und die gesundheitliche Wahrnehmung der Patienten zu fördern, um einen patientenseitigen Einbezug in informierte Entscheidungsprozesse zur medizinischen Versorgung gewährleisten zu können. Dies steht im Einklang mit der im *Nationalen Aktionsplan Gesundheitskompetenz* formulierten Zielsetzung, die Teilhabe aller Bürger durch eine systematische und bundesweite Förderung der GK zu stärken und bestehende Ungleichheiten zu verringern [17]. Orientierung können vorhandene Leitlinien und Materialsammlungen zur Förderung der GK bieten [19], die um notaufnahmespezifische Aspekte zu ergänzen sind.

## Fazit für die Praxis

- Die GK beeinflusst, wie Patienten die Notfallversorgung in Deutschland nutzen.
- Die GK erwies sich in der vorliegenden Stichprobe als eigenständiger Indikator für das Notfallverhalten, weitgehend unabhängig von Geschlecht, Bildung und Migrationshintergrund.
- Patienten mit niedriger GK haben häufig bereits länger anhaltende Symptome und schätzen ihre Behandlungsdringlichkeit als höher ein.
- Die Einbindung von hausärztlicher Versorgung und Notfallversorgung unterscheidet sich in Abhängigkeit von der GK der Patienten. Bei Patienten mit niedriger GK wurde häufiger der Hausarzt bereits wegen der Symptome konsultiert.

## Footnotes

1 Russisch und Türkisch sind die am häufigsten benötigten Fremdsprachen im betrachteten Klinikum. Englisch wird indes, nach langjähriger Erfahrung des Klinikums, nur sehr selten benötigt und wurde deshalb nicht integriert.

## References

[1] Bauer SE, Schumacher JR, Hall AG (2016). Primary care experiences of emergency department patients with limited health literacy. J Ambul Care Manage.

[2] Boender TS, Greiner F, Kocher T (2020). Inanspruchnahme deutscher Notaufnahmen während der COVID-19-Pandemie – der Notaufnahme-Situationsreport (SitRep). Epidemiol Bull.

[3] Bundesministerium für Gesundheit (2020) Reform der Notfallversorgung. https://www.bundesgesundheitsministerium.de/notfallversorgung.html. Zugegriffen: 1. Dez. 2020

[4] Greene JC, Haun JN, French DD (2019). Reduced hospitalizations, emergency room visits, and costs associated with a web-based health literacy, aligned-incentive intervention: mixed methods study. J Med Internet Res.

[5] Griffey RT, Kennedy SK, D’Agostino McGowan L (2014). Is low health literacy associated with increased emergency department utilization and recidivism?. Acad Emerg Med.

[6] Grossmann F, Delport K, Kellert D (2009). Emergency Severity Index. Deutsche Übersetzung eines validen Triageinstruments. Notfall Rettungsmed.

[7] Hajiloueian E (2011) Inanspruchnahme von Notfallambulanzen in Berlin in den Jahren 2006/2007: Einfluss von Geschlecht, Alter, Bildungsgrad und ethnischer Herkunft. https://refubium.fu-berlin.de/handle/fub188/2230. Zugegriffen: 21. Nov. 2019

[8] HLS-EU Consortium (2015) Comparative report of health literacy in eight EU member states. The European Health Literacy Survey HLS-EU 2012. https://cdn1.sph.harvard.edu/wp-content/uploads/sites/135/2015/09/neu_rev_hls-eu_report_2015_05_13_lit.pdf. Zugegriffen: 21. Nov. 2019

[9] Jordan S, Hoebel J (2015). Gesundheitskompetenz von Erwachsenen in Deutschland. Bundesgesundheitsblatt Gesundheitsforschung Gesundheitsschutz.

[10] Mazurik L, Javidan AP, Higginson I (2020). Early lessons from COVID-19 that may reduce future emergency department crowding. Emerg Med Australas.

[11] Messerle R, Appelrath M (2018). Die Zukunft der Notfallversorgung in Deutschland. Urologe.

[12] Miller TA (2017). Health literacy and adherence to medical treatment in chronic and acute illness: a meta-analysis. Patient Educ Couns.

[13] O’Dowd A (2020). Emergency departments must not return to pre-covid days of overcrowding and lack of safety, says college. BMJ.

[14] Ramshorn-Zimmer A, Schröder R, Fakler J (2020). Notaufnahme während der Corona-Pandemie: Weniger Non-COVID-19-Notfälle. Dtsch Arztebl.

[15] Röthlin F, Pelikan JM, Ganahl K (2013) Die Gesundheitskompetenz der 15-jährigen Jugendlichen in Österreich. Abschlussbericht der österreichischen Gesundheitskompetenz Jugendstudie im Auftrag des Hauptverbands der österreichischen Sozialversicherungsträger (HVSV). http://www.hauptverband.at/cdscontent/load?contentid=10008.597350&version=1395738807. Zugegriffen: 21. Nov. 2019

[16] Schäffer D, Berens E-M, Vogt D (2017). Gesundheitskompetenz der Bevölkerung in Deutschland. Dtsch Arztebl.

[17] Schäffer D, Hurrelmann K, Bauer U (2018). Nationaler Aktionsplan Gesundheitskompetenz. Die Gesundheitskompetenz in Deutschland stärken.

[18] Scherer M, Lühmann D, Kazek A (2017). Patienten in Notfallambulanzen. Querschnittstudie zur subjektiv empfundenen Behandlungsdringlichkeit und zu den Motiven die Notfallambulanzen von Krankenhäusern aufzusuchen. Dtsch Arztebl.

[19] Schmidt-Kaehler S, Vogt D, Berens EM (2017). Gesundheitskompetenz. Verständlich informieren und beraten. Material- und Methodensammlung zur Verbraucher- und Patientenberatung für Zielgruppen mit geringer Gesundheitskompetenz.

[20] Schmiedhofer M, Möckel M, Slagman A (2016). Patient motives behind low-acuity visits to the emergency department in Germany: a qualitative study comparing urban and rural sites. BMJ Open.

[21] Schmiedhofer M, Möckel M, Slagman A (2017). Inanspruchnahme zentraler Notaufnahmen: Qualitative Erhebung der Motivation von Patientinnen und Patienten mit nichtdringlichem Behandlungsbedarf. Gesundheitswesen.

[22] Schroedter J, Lechert Y, Lüttiger P (2006). Die Umsetzung der Bildungsskala ISCED-1997 für die Volkszählung 1970, die Mikrozensus-Zusatzerhebung 1971 und die Mikrozensen 1976–2004. ZUMA-Methodenbericht.

[23] Searle J, Muller R, Slagman A (2015). Überfüllung der Notaufnahmen. Gründe und populationsbezogene Einflussfaktoren. Notfall Rettungsmed.

[24] Slagman A, Behringer W, Greiner F (2020). Medical emergencies during the COVID-19 pandemic. Dtsch Arztebl Int.

[25] Statistisches Bundesamt (2017) Altersaufbau 2017. https://service.destatis.de/bevoelkerungspyramide/index.html#!y=2017&v=2. Zugegriffen: 21. Nov. 2019

[26] Statistisches Bundesamt (2019) Bevölkerung im Alter von 15 Jahren und mehr nach allgemeinen und beruflichen Bildungsabschlüssen nach Jahren. https://www.destatis.de/DE/Themen/Gesellschaft-Umwelt/Bildung-Forschung-Kultur/Bildungsstand/Tabellen/bildungsabschluss.html. Zugegriffen: 21. Nov. 2019

[27] Tschaikowsky T, Becker von Rose A, Consalvo S (2020). Patientenzahlen im Rahmen der COVID-19-Pandemie in einer zentralen Notaufnahme. Notfall Rettungsmed.

